# Continuous positive airway pressure increases CSF flow and glymphatic transport

**DOI:** 10.1172/jci.insight.170270

**Published:** 2023-06-22

**Authors:** Burhan Ozturk, Sunil Koundal, Ehab Al Bizri, Xinan Chen, Zachary Gursky, Feng Dai, Andrew Lim, Paul Heerdt, Jonathan Kipnis, Allen Tannenbaum, Hedok Lee, Helene Benveniste

**Affiliations:** 1 Department of Anesthesiology, Yale School of Medicine, New Haven, Connecticut, USA.; 2 Department of Anesthesiology, Renaissance School of Medicine, Stony Brook University, Stony Brook, New York, USA.; 3 Department of Medical Physics, Memorial Sloan Kettering Cancer Center, New York City, New York, USA.; 4 Quantitative Data Sciences, Global Product Development Pfizer Inc., Groton, Connecticut, USA.; 5 Department of Medicine, University of Toronto, Toronto, Canada.; 6 Brain Immunology and Glia (BIG) Center, Department of Pathology and Immunology, Washington University School of Medicine, St. Louis, Missouri, USA.; 7 Departments of Computer Science and Applied Mathematics & Statistics, College of Engineering and Applied Sciences, Stony Brook University, Stony Brook, New York, USA.; 8 Department of Biomedical Engineering, Yale School of Medicine, New Haven, Connecticut, USA.

**Keywords:** Neuroscience, Anesthesiology, Neurological disorders, Respiration

## Abstract

Respiration can positively influence cerebrospinal fluid (CSF) flow in the brain, yet its effects on central nervous system (CNS) fluid homeostasis, including waste clearance function via glymphatic and meningeal lymphatic systems, remain unclear. Here, we investigated the effect of supporting respiratory function via continuous positive airway pressure (CPAP) on glymphatic-lymphatic function in spontaneously breathing anesthetized rodents. To do this, we used a systems approach combining engineering, MRI, computational fluid dynamics analysis, and physiological testing. We first designed a nasal CPAP device for use in the rat and demonstrated that it functioned similarly to clinical devices, as evidenced by its ability to open the upper airway, augment end-expiratory lung volume, and improve arterial oxygenation. We further showed that CPAP increased CSF flow speed at the skull base and augmented glymphatic transport regionally. The CPAP-induced augmented CSF flow speed was associated with an increase in intracranial pressure (ICP), including the ICP waveform pulse amplitude. We suggest that the augmented pulse amplitude with CPAP underlies the increase in CSF bulk flow and glymphatic transport. Our results provide insights into the functional crosstalk at the pulmonary-CSF interface and suggest that CPAP might have therapeutic benefit for sustaining glymphatic-lymphatic function.

## Introduction

The brain’s network of perivascular channels, which exists between the vascular endothelium and glial end feet with aquaporin 4 water channels, is referred to as the glymphatic system and facilitates the exchange of cerebrospinal fluid (CSF) with interstitial fluid (ISF) for waste removal via perivenous channels (1, 2). Functionally, the glymphatic system interconnects with the meningeal lymphatics for clearance of waste solutes to the cervical lymph nodes and systemic circulation for ultimate breakdown (3–5). The importance of the glymphatic and lymphatic systems in maintaining brain health is strongly supported by studies demonstrating that the 2 systems clear soluble amyloid-β (Aβ) (1, 6) and tau (7). Moreover, glymphatic transport has been observed to decline in transgenic mouse models of Alzheimer’s disease (AD) (8) and cerebral small vessel disease (9–12), including cerebral amyloid angiopathy (13). Likewise, ablation of meningeal lymphatic vessels increases Aβ deposition and inflammation in the meninges and brains of transgenic AD mice (3). Collectively, all of these studies highlight the therapeutic potential of sustaining or augmenting glymphatic-lymphatic system function in order to prevent cognitive decline and neurodegeneration.

Glymphatic waste removal is dependent on brain state, and in rodents, clearance of soluble Aβ increases during slow-wave sleep and with anesthetic regimens producing electroencephalographic slow-wave activity when compared with wakefulness (14–16). Analogous studies in humans recently supported the glymphatic system’s dependency on brain state by showing that solute clearance decreased with sleep deprivation (17). Several key physiological regulators, including cardiovascular pulsatility (11, 18, 19), vasomotion (20, 21) and respiratory function (22), can also influence CSF flow, perivascular solute transport, and brain waste clearance. Studies in humans have shown that a deep inspiratory breath (so-called diaphragmatic breathing or deep breathing) significantly enhances upward CSF movement toward the brain in a manner counterbalanced by blood leaving the skull via draining veins, including the epidural venous plexuses of the upper cervical spine (23). It has been assumed that the increase in cephalad CSF flow with deep breathing likely invigorates CSF-ISF exchange and brain waste clearance (23, 24). The effects of sleep and other physiological regulators on glymphatic function have inspired investigations into how physical maneuvers might enhance brain waste clearance. However, because it is technically challenging to test the effect of a deep breath during the respiratory cycle on glymphatic-lymphatic system function in spontaneously breathing rodents or humans, the effect of such exercises has not yet been thoroughly explored. While we cannot implement a voluntary deep breathing scenario for rodent studies similar to clinical experiments (22, 25, 26), we instead investigated the effect of augmenting lung function via continuous positive airway pressure (CPAP) on glymphatic-lymphatic function in anesthetized, spontaneously breathing rats. In the clinical arena, CPAP works by delivering a continuous pressure during inspiration as well as expiration, which increases the upper airway volume and the functional residual capacity (FRC), thereby opening collapsed alveoli, decreasing pulmonary shunt, and improving oxygenation (27–29). CPAP is effective in treating obstructive sleep apnea (OSA) (30) and maintaining airway patency in anesthetized/sedated spontaneously breathing individuals (31). We hypothesized that the application of CPAP would (a) improve respiratory function including FRC in spontaneously breathing anesthetized rats and (b) augment CSF flow dynamics and secondarily glymphatic-lymphatic transport. To test our hypotheses, we first designed and constructed an MRI-compatible nasal CPAP device for use in anesthetized rats. We then conducted a multifaceted investigation to dissect physiological changes with CPAP and impact on CSF flow dynamics and glymphatic transport as measured by dynamic contrast-enhanced magnetic resonance imaging (DCE-MRI) (32). Specifically, our systems approach combined 3D computer-aided design processes, MRI, computational fluid dynamics analysis based on regularized optimal mass transport (rOMT) (13), and in vivo physiology.

We found that CPAP set at 3 cmH_2_O opened the upper airway, significantly increased the FRC, and improved arterial oxygenation in anesthetized and spontaneously breathing rats. We also demonstrated that CPAP subtly raised intracranial pressure (ICP) and the ICP waveform pulse amplitude. The CPAP-induced physiological changes were associated with increased CSF flow speed and locally enhanced glymphatic transport to several brain regions, including the dorsal hippocampus and cortex. Moreover, CPAP also sustained drainage to the deep cervical lymph nodes. We suggest that the mechanism behind the effect of CPAP on CSF flow is related to the CPAP-induced increase in the ICP waveform pulse amplitude, which augments CSF bulk flow during spontaneous breathing. Our results provide insights into the functional crosstalk at the pulmonary-CSF interface and suggest that CPAP augments CSF flow and glymphatic transport, which might have therapeutic implications for prevention of neurodegenerative disorders.

## Results

### CPAP chamber design.

**Figure 1:**
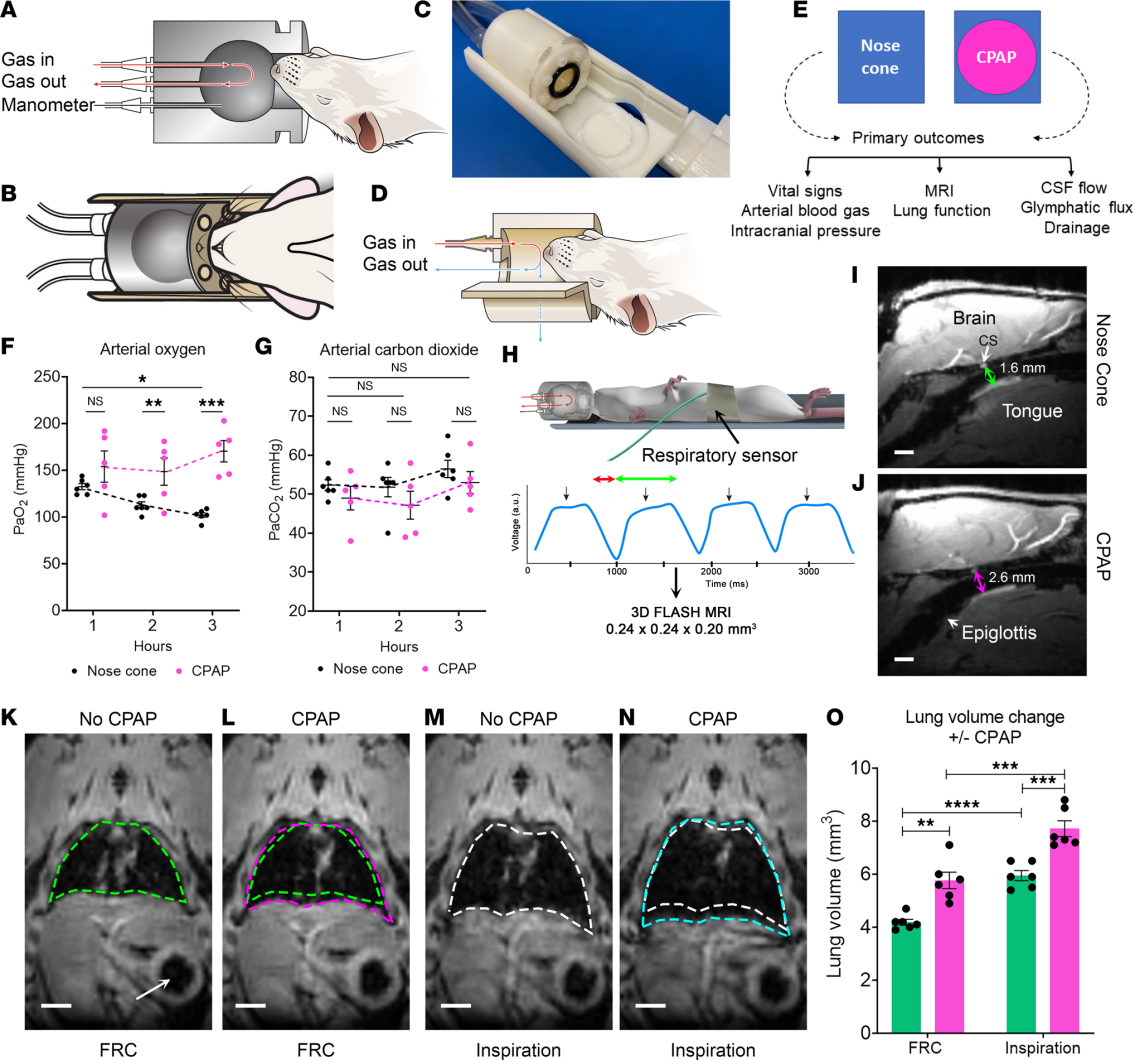
Nasal CPAP adapted for use in rats functions similarly to clinical CPAP. (**A**–**D**) CPAP chamber and nose cone. (**E**) Overview of experiments. (**F**) Graph with arterial oxygenation values (PaO_2_) in rats breathing via the nose cone (*n* = 6, black dots) and CPAP (*n* = 5, pink dots). Each dot represents values from 1 rat. Data are shown as mean ± SEM. A linear mixed model with fixed effects of time (blood draw time), group (no CPAP vs. CPAP), and the interaction between time and group. *P = 0.029, **P = 0.028, ***P < 0.001. (**G**) Corresponding graph with arterial carbon dioxide (PaCO_2_) values. (**H**) Overview of experimental design for lung studies. The inhalation and exhalation phases are indicated with a red and green arrow, respectively. Black arrows indicate respiratory triggers. FLASH, fast low angle shot sequence. (**I** and **J**) Anatomical MRIs in the midbrain sagittal-view plane, which captures the upper airway anatomy including the tongue, epiglottis, and trachea. Arrows represent distance from the cavernous sinus (CS, white arrow) to the base of the tongue in a rat breathing via the nose cone (green arrow, distance = 1.6 mm) and with CPAP (pink arrow, distance = 2.6 mm). Scale bar = 3 mm. (**K** and **L**) Outline of the lungs at end expiration (functional residual capacity, FRC) without (green contour) or with CPAP (pink contour). (**M** and **N**) Corresponding MRI images acquired at peak inspiration with and without CPAP. The arrow in **K** points to the stomach. Scale bars = 500 μm. (**O**) Graph with values of FRC and inspiratory lung volumes from 6 rats where the rats served as their own controls, obtained with no CPAP (green bars) and with CPAP (pink bars). Data are mean ± SEM. A linear mixed models with fixed effects of time (respiratory cycle, i.e., end expiration and inspiration), group (no CPAP vs. CPAP), and the interaction between respiratory cycle and group. **P = 0.003, ***P = 0.001, ****P < 0.001.

Therapeutic CPAP devices for use in humans are designed with a wide variety of mask interfaces. Examples include nasal, oral, or combined oronasal masks, and clinical studies suggest that nasal interfaces are generally better tolerated (33). Like human neonates, rats are obligate nose breathers, and we therefore designed the chamber as a nasal CPAP interface. To design the best possible device for the studies, we first tested different CPAP chamber interface designs (e.g., variable nasal/snout configurations) on the bench in rats anesthetized with a balanced anesthesia consisting of dexmedetomidine and low-dose isoflurane (DEXM-I) (13). Our definition of “best design” was a CPAP device chamber that (a) was adapted with a mask interface that reliably allowed for the rat’s snout to rest inside the pressurized chamber while holding a tight seal for delivery of airflow down the nasal away, (b) was combined with an outlet for chamber pressure readings during studies, and (c) was MRI compatible and small enough to fit into the custom-designed MRI animal holder with the radiofrequency (RF) coil. The design process was adapted in parallel with studies titrating and defining the optimal CPAP chamber pressure as guided by physiological parameters and MRI-defined lung volume changes captured in the anesthetized rats during spontaneous breathing. The final nasal CPAP device design is shown in Figure 1, A–C, and Supplemental Figure 1; supplemental material available online with this article; https://doi.org/10.1172/jci.insight.170270DS1 The CPAP chamber was constructed as a sphere connected to a small cylindrical extension, adapted with a rubber O-ring, which served as a seal between the chamber and rat’s snout (Figure 1, A–C). Note that the CPAP chamber was fitted inside a cylinder for easy and sturdy positioning within the MRI animal holder (Figure 1, A and B). During experiments, air and O_2_ (fraction of inspired oxygen, FiO_2_, of 30%–40%) mixed with isoflurane were delivered into the CPAP chamber at a flow speed of 1 L/min. One of the chamber outlet ports was connected to a needle valve, which enabled fine adjustment of the chamber pressure by restricting the gas outflow, thereby building back pressure inside the chamber. An analog manometer was connected downstream to continuously monitor the CPAP chamber pressure during experiments (Supplemental Figure 1). Control animals received similar airflow and anesthesia via a nonpressurized nose cone (Figure 1D).

### Effect of CPAP on arterial blood gases and blood pressure.

We first conducted pilot experiments in rats to visually observe the effect of applying CPAP on the respiratory pattern and respiratory rate in real time while titrating chamber pressures between 1 and 5 cmH_2_O. We discovered that CPAP pressures over 4 cmH_2_O were not well tolerated as the rats exhibited paradoxical labored breathing and prolonged apneic episodes similar to what has been reported in neonates (8). However, CPAP chamber pressures up to 3 cmH_2_O implemented by gradually increasing the chamber pressure in small 0.5 cmH_2_O increments every 5 minutes was well tolerated, and no paradoxical breathing or apneic episodes were observed. Having established the CPAP pressure implementation protocol, we prospectively studied physiological parameters over a 3-hour experimental time period in anesthetized rats breathing spontaneously via the nose cone in comparison to rats breathing with 3 cmH_2_O CPAP (Figure 1E). Analysis of arterial blood gas measurements revealed that the arterial oxygenation (PaO_2_) decreased by approximately 20% over time in rats breathing via the nose cone, while in rats breathing CPAP, PaO_2_ improved by approximately 10% (Figure 1F and Supplemental Table 1). There were no differences in arterial carbon dioxide (PaCO_2_) levels over time (Figure 1G). Likewise, arterial pH and arterial blood pressure were similar across groups (Supplemental Table 1). We also extracted the pulse pressure from the femoral artery waveforms from each rat, and the mean pulse pressure over the 3-hour experimental period was similar across the 2 groups (*P* value = 0.1328).

### CPAP expands the upper airway and increases FRC in anesthetized rats.

Clinical data have demonstrated that CPAP, by exerting a constant and continuous positive pressure throughout the respiratory cycle, opens the upper airway and prevents dynamic collapse (29, 34). Moreover, in humans, CPAP is also known to increase the FRC in a manner dependent on the CPAP pressure settings (35, 36). To establish that our CPAP device for rats operated similarly to clinical CPAP, we first assessed upper airway patency from the 2 groups. Anatomical MRIs displayed in the midsagittal plane view that captures the upper airway, tongue, and trachea are shown from a rat breathing via nose cone (Figure 1I) and with CPAP (Figure 1J). It is evident that the upper airway distance measured from the cavernous sinus at the skull base to the root of the tongue (arrows) is shorter in the rat breathing via the nose cone compared with CPAP, suggesting partial collapse of the upper airway in nose cone rats due to the effect of anesthesia on airway muscle tone. Indeed, the upper airway diameter (posterior-to-anterior direction) from cavernous sinus to tongue (Figure 1, I and J) was approximately 35% larger in rats breathing with CPAP compared with nose cone (CPAP [*n* = 8] mean diameter = 2.5 ± 0.1 mm vs. controls [*n* = 8] mean diameter = 1.9 ± 0.1 mm, mean difference = –0.6mm, 95% CI [–0.9, –0.3 mm], *P* value = 0.001). To further establish that our CPAP device for rats mimicked clinical CPAP, we implemented respiratory-gated 3D MRI during the respiratory cycle to track lung volume changes (Figure 1H and Supplemental Figure 2). In these experiments, the rats served as their own controls and underwent 2 scans with or without CPAP with the order counterbalanced. CPAP was implemented gradually over a 30-minute period, and scanning started when the CPAP pressure reached 3 cmH_2_O. Guided by the respiratory signal recorded during imaging, we collected and measured lung volumes at the end of expiration (representing FRC) and at the peak of inspiratory effort. FRC lung volumes from a rat breathing spontaneously with and without CPAP are outlined in Figure 1, K and L. The increase in the FRC with CPAP (pink contour) compared with no CPAP (green contour) is illustrated in Figure 1L and shows that FRC increases primarily involved the lung bases, similar to what has been observed in humans (29, 36). CPAP also increased the peak inspiratory volume (defined as lung volume measured at peak inspiration) when compared with no CPAP (Figure 1, M and N). Specifically, CPAP at 3 cmH_2_O pressure increased FRC by 27% and peak inspiratory volume by approximately 22% (Figure 1O). There were no significant differences in tidal volume (defined as the difference between inspiratory volume and FRC) or minute ventilation across the rats breathing spontaneously with or without CPAP (Supplemental Figure 3). Note that the stomach was also in the field of view on the lung MRI images (white arrow, Figure 1K) and showed no apparent increase in gastric volume with CPAP. From these data we concluded that our CPAP device designed for the rats set at 3 cmH_2_O functioned similarly to clinical devices as evidenced by an opening of the upper airway, an increase in the FRC, as well as improved oxygenation noted from our bench experiments.

### CPAP is associated with increases in CSF solute speed and local glymphatic transport.

**Figure 2:**
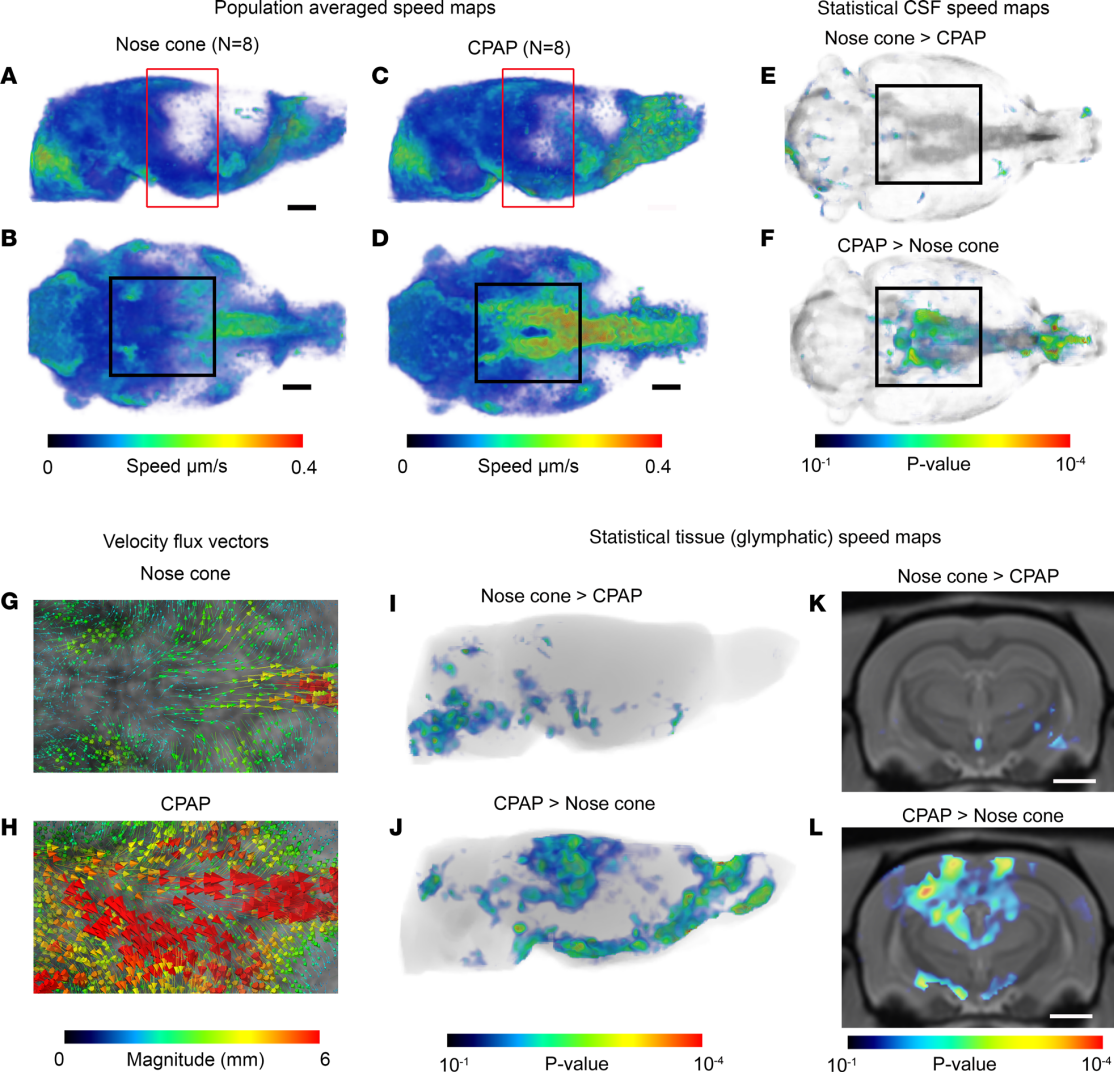
CPAP increases CSF flow speed and glymphatic transport locally. (**A** and **B**) Spatially normalized population-averaged whole-brain speed maps of rats (*n* = 8) breathing with nose cone shown in 2 orthogonal-plane views. (**C** and **D**) Corresponding speed maps of the CPAP group (*n* = 8) showing fast speed trajectories along the circle of Willis area when compared with the nose cone group (black boxes in **B** and **D**). The red-colored boxes in **A** and **C** indicate the midbrain area where glymphatic transport also appears different across groups. Scale bars = 2 mm. (**E** and **F**) Statistical maps (color coded for *P* values) overlaid onto a CSF compartment binary map highlighting voxel areas with higher CSF speed for the 2 conditions: NC > CPAP (**E**) and CPAP > NC (**F**). The black boxes highlight the circle of Willis. NC, nose cone. (**G** and **H**) Representative examples of velocity flux vectors (color coded for magnitude) shown from the ventral surface of the brain from a rat breathing via nose cone or with CPAP. (**I** and **J**) Statistical maps (color coded for *P* values) for tissue solute speed and flux (representing glymphatic transport) highlighting local areas with higher glymphatic transport and solute speed for the 2 conditions: NC > CPAP (**I**) and CPAP > NC (**J**). (**K** and **L**) Statistical maps (color coded for *P* values) for glymphatic transport overlaid onto an anatomical brain map highlighting voxel areas in the dorsal hippocampus with increased glymphatic speed/flux for the condition CPAP > NC (**L**). Scale bars = 3 mm. The voxel-wise statistical analysis was performed using an independent 2-sample *t* test in the framework of general linear modeling.

To test the hypothesis that CPAP will augment CSF flow dynamics and secondarily alter glymphatic transport, we performed DCE-MRI with intrathecal gadoteric acid (Gd-DOTA) in rats exposed to either nose cone or CPAP breathing. The DCE-MRI data were analyzed using our previously developed computational framework based on rOMT theory (10, 37) optimized for measurement of CSF flow speed and glymphatic transport (14, 38). Briefly, from DCE-MRI whole-brain data, the rOMT analysis derives trajectories of fluid and solute movement — known as “pathlines” — as well as important properties such as solute speed along these pathlines over a 100-minute tracer circulation time (for more details, see Chen et al., ref. 13). We first generated 3D whole-brain pathline maps color coded for speed from the rats breathing via the nose cone or CPAP device. The speed maps represent solute and fluid transport across the whole brain and can be further used to extract the mean solute speed and total volume of solute transport (v-flux) within the CSF and tissue compartments or regionally. Note that v-flux of the brain tissue compartment represents “glymphatic transport.” Population-averaged whole-brain speed maps of nose cone and CPAP rats in sagittal- and ventral-view planes are shown in Figure 2, A–D. The maps revealed that CSF solute speed on the ventral surface near the skull base was increased with CPAP (Figure 2D, black box) when compared with rats breathing via nose cone (Figure 2B, black box). Corresponding statistical parametric maps (color coded for *P* values) verified higher CSF solute speed at the ventral surface in CPAP rats (Figure 2, E and F, black boxes). Post hoc regional analysis at the circle of Willis revealed that CSF solute speed was approximately 50% greater in the CPAP rats when compared with nose cone breathing (CPAP circle of Willis solute speed = 0.41 ± 0.07 μm/s vs. control circle of Willis solute speed = 0.21 ± 0.03 μm/s, mean difference = –0.20 μm/s, 95% CI [–0.37, –0.03 μm/s], *P* value = 0.023). The rOMT methodology also derives velocity flux vectors, which inform on the magnitude and direction of the fluid streams (13). In rats breathing via the nose cone, the velocity flux vectors on the ventral surface were distributed in a symmetric pattern around the midline and pointed toward the tissue bed (Figure 2G). In CPAP rats, the corresponding velocity flux vectors were of higher magnitude (red arrows, Figure 2H), in agreement with higher CSF solute speed in this area. Previously, in a rat model of cerebral amyloid angiopathy, we reported that high-speed CSF flow at the skull base was associated with high-magnitude velocity flux vectors and decreased glymphatic transport (13). However, in CPAP rats, the high-magnitude velocity flux vectors were directed toward the tissue bed and did not negatively influence glymphatic transport. Indeed, the whole-brain speed maps suggested overall more glymphatic transport activity into the midbrain area in the CPAP compared with nose cone rats (red-colored boxes in Figure 2, A and C). Corresponding statistical parametric maps (color coded for *P* values) verified that while midbrain and olfactory bulb areas were associated with increased glymphatic transport activity in CPAP rats (Figure 2I), the brain stem and pons exhibited more glymphatic transport activity in the nose cone rats (Figure 2J). Images in Figure 2, K and L, showed significantly more glymphatic transport activity in the dorsal hippocampus and thalamus with CPAP when compared with rats breathing via the nose cone. More anatomical details of regional differences in glymphatic transport across the 2 groups can be found in Supplemental Figure 4. In sum, from these data, we concluded that CPAP augmented CSF solute speed along the skull base and redistributed glymphatic transport to the midbrain when compared with rats breathing via the nose cone.

### Is CPAP influencing known drivers of CSF and glymphatic transport?

**Figure 3:**
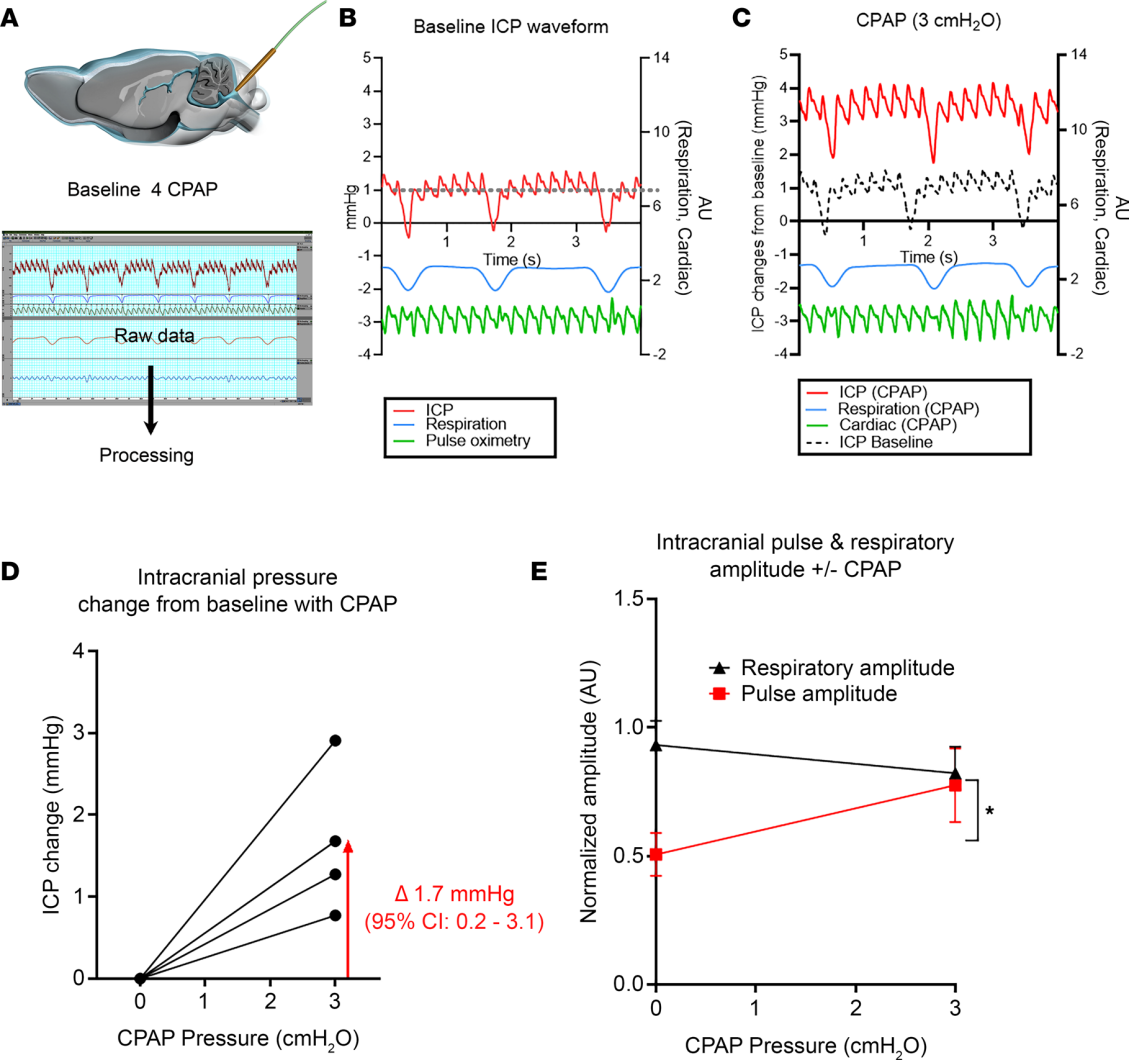
CPAP induces an increase in ICP and pulse pressure amplitude. (**A**) Schematic illustration of setup. (**B**) Representative ICP waveform recorded from the pressure probe at baseline showing the pulse and respiratory oscillations on the ICP waveform (red) with parallel recording of respiration (blue) and HR via pulse oximetry (green). The gray dashed line indicates the baseline ICP opening pressure approximately 1 mmHg recorded from a separate rat not undergoing CPAP. (**C**) Corresponding ICP waveforms from the rat shown in **B**, while breathing with a CPAP pressure of 3 cmH_2_O. The average ICP pressure and pulse amplitude are noticeably increased with the applied CPAP pressure. (**D**) Increase in the mean ICP pressure from baseline with application of CPAP from *n* = 4 rats (1 rat was excluded due to baseline drifting during the experiment). (**E**) Comparison of changes from baseline in the mean respiratory amplitude and mean pulse amplitude (normalized to baseline) with CPAP. An independent *t* test revealed that there were no differences in the respiratory amplitude with CPAP; however, the arterial pulse amplitude increased significantly from baseline with CPAP (*P = 0.0315).

Our data showed that CPAP augmented CSF flow speed and glymphatic transport locally, and we next explored potential underlying physiological drivers of these effects. It is well known that increases in systemic cardiovascular pulsatility, i.e., heart rate (HR), can enhance glymphatic influx and perivascular transport in normal rats (11, 19). We therefore analyzed and compared HR differences across the CPAP and nose cone groups, and this analysis revealed an approximately 7% higher mean HR of CPAP compared with nose cone rats (CPAP [*n* = 9] HR = 253 ± 12 beats per minute, bpm, vs. nose cone [*n* = 8] HR = 236 ± 17 bpm, mean difference = –17 bpm, 95% CI [–33, –1] bpm, *P* value = 0.037). There were no differences in the mean respiratory rate (RR) across the 2 groups as measured during the DCE-MRI experiments (CPAP [*n* = 9] RR = 44 ± 2 bpm vs. nose cone [*n* = 8] RR = 43 ± 3 bpm, mean difference = –1 bpm, 95% CI [–8, 7] bpm, *P* value = 0.842). Given that CPAP pressurizes the airway and the lungs, it likely also elevates ICP. Accordingly, we tested the effect of CPAP on ICP in experiments performed in separate series of DEXM-I–anesthetized rats. The ICP measurements were made via a small catheter positioned in the cisterna magna (Figure 3A), and correct placement was verified by the presence of an ICP waveform with pulse and respiratory oscillations (Figure 3, B and C). The mean ICP increased from baseline to about 1.7 mmHg at the final 3 cmH_2_O CPAP pressure (Figure 3D). We also calculated the respiratory and pulse pressure amplitudes from the ICP waveforms and documented no significant change in the respiratory amplitude from baseline to CPAP; however, a significant approximately 40% increase from baseline was observed in the pulse pressure amplitude with the implementation of CPAP (Figure 3E). From these data, we concluded that 3 cmH_2_O CPAP was associated with an increase in the mean ICP and significant augmentation of the pulse pressure amplitude as measured via the ICP waveform when compared to baseline.

### CPAP sustains drainage to cervical lymph nodes.

**Figure 4:**
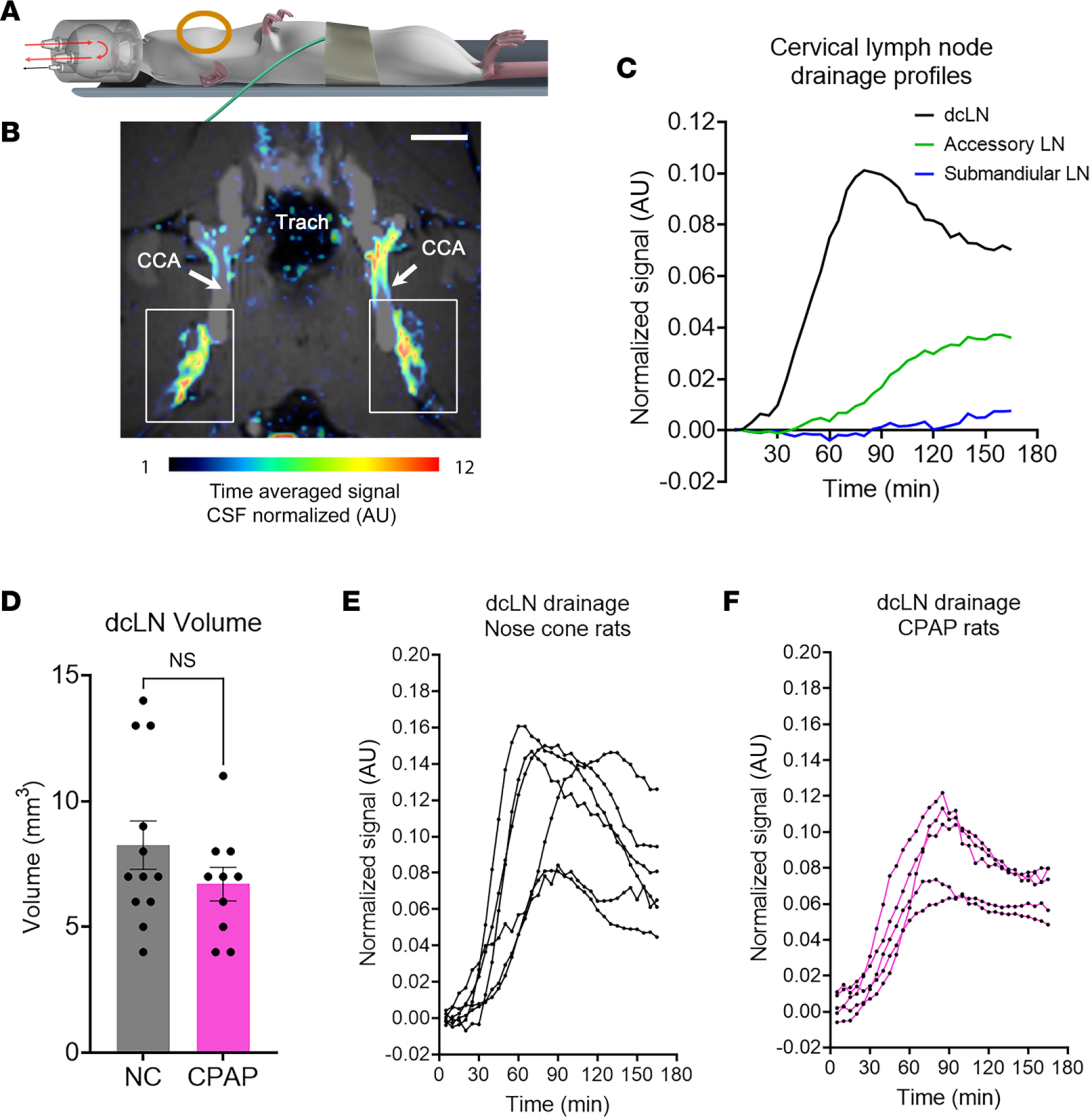
CPAP sustains drainage to the cervical lymph nodes. (**A**) Illustration of the radiofrequency (RF) surface coil positioned above the neck of the rat in supine position while breathing with CPAP. (**B**) Anatomical MRI image at the level of the deep cervical lymph nodes (dcLN, white boxes) overlaid with corresponding solute drainage map represented by color coded signal intensity normalized to the CSF signal and time-averaged over approximately 2 hours. Scale bars = 2.5 mm. Trach, trachea; CCA, common carotid artery. (**C**) Time signal curves of tracer uptake in dcLNs (shown in black), accessory lymph nodes (green), and submandibular lymph nodes (blue) from a normal rat. (**D**) Volumes of the dcLNs extracted from rats breathing via the nose cone (gray bar) or CPAP set at 3 cmH_2_O (pink bar). Each dot above the bars represents values from 1 dcLN with 2 dcLNs/rat. Data are mean ± SEM. An independent 2-sided *t* test was used and revealed no differences in dcLN volume across the 2 groups (*P* = 0.2016). (**E**) Time signal curves (TSC) from the dcLNs of each rat derived from independent experiments of *n* = 6 rats breathing via the nose cone. Each line represents average signal change of the right- or left-sided dcLNs from each rat. (**F**) Corresponding TSC data extracted from dcLNs derived from independent experiments of *n* = 5 rats breathing with CPAP set at 3 cmH_2_O.

Research reports suggest that glymphatic waste egress from the brain is critically dependent on connections to the meningeal lymphatics (3, 39, 40). Specifically, egressing fluids and waste solutes from the brain parenchyma are thought to pass via “hotspots” to the meningeal lymphatics (4, 41). The meningeal lymphatics travel along the dural blood vasculature, in particular along large dural veins, including the cavernous vein, and connect via afferent lymphatics to the cervical lymph nodes (41). However, CSF can also exit along cranial nerves, including the olfactory nerves, passing through the cribriform plate into a submucosal lymphatic network in the nasal cavity that drains to superficial lymph nodes as well as draining cervical lymph nodes (dcLNs) (3, 41). Based on our results demonstrating enhanced CSF flow and regional augmentation of glymphatic transport with CPAP, we predicted that drainage to the dcLNs would be sustained with CPAP when compared with rats breathing via the nose cone. We previously developed a DCE-MRI–based technique to track a Gd-tagged tracer (GadoSpin-P) from the CSF and brain to the cervical lymph nodes (13). For these studies, the RF coil was positioned above the rat’s neck region (Figure 4A), and the MRI field of view captured all the cervical lymph nodes, including the dcLNs (Figure 4B). We reapplied this method to track GadoSpin-P kinetics at the level of the cervical lymph nodes in rats breathing via the regular nose cone or CPAP device (Figure 4C). The volume of the dcLNs was similar across the groups (Figure 4D). From the time signal curves (TSCs), it was evident that the dcLNs of both nose cone and CPAP rats drained GadoSpin-P (Figure 4, E and F). The mean TSC time to peak of the dcLNs was similar across the groups (nose cone rats [*n* = 6] dcLN peak time: 87.5 ± 9.8 minutes vs. CPAP rats [*n* = 5] dcLN peak time 87.0 ± 3.3 minutes, mean difference = 0.5 minutes, 95% CI [–24.7, 25.7] minutes, *P* value = 0.963). The mean peak signal magnitude (normalized to CSF signal intensity) was also similar across the groups (nose cone rats [*n* = 6] dcLN peak magnitude: 0.13 ± 0.02 AU vs. CPAP rats [*n* = 5] dcLN peak magnitude 0.09 ± 0.01 AU mean difference = 0.04 AU, 95% CI [–0.01, 0.08] AU, *P* value = 0.092). The accessory and submandibular lymph nodes were also analyzed, and the TSCs for most of these nodes showed a steady increase over the 3-hour experimental time window. The drainage capacity of the accessory lymph nodes was measured as AUC and was similar across the 2 groups (*P* value = 0.112).

## Discussion

The function of the glymphatic and meningeal lymphatic systems for brain waste clearance declines with aging (42, 43). Moreover, several animal as well as human studies have shown that brain waste clearance is reduced in AD (3, 8), vascular dementia (10), normal pressure hydrocephalus (44), and Parkinson’s disease (45). This accumulating evidence has spurred on new clinical trials that now implement primary outcomes metrics such as glymphatic flow and lymphatic drainage to investigate the drivers and function of the glymphatic-lymphatic system (46–48). Many physiological regulators and positional maneuvers are known to alter fluid dynamics in the brain, including cardiovascular contractility (11, 19, 24), deep breathing (22, 23, 25), body posture (49), exercise (50), and sleep/wake cycle (16, 24, 51, 52). In particular, the effects of sleep on glymphatic function have inspired new clinical trials focused on the potential beneficial effects of sleep and meditative states on glymphatic flow in the human brain (53) as well as the negative impact of sleep disruption in patients with OSA on cognitive function. Although the majority of studies involving sleep and brain health are observational, there is increasing evidence that patients with OSA who are compliant with their CPAP regimen have better outcomes in terms of cognition and decreases in biomarkers of AD progression (54–58). Several mechanisms have been proposed to explain these improvements in patients with OSA, such as decreased arousal and fragmentation of sleep and improved oxygenation. However, no in-depth studies on the physiological effects of CPAP on CSF and glymphatic flow dynamics have been performed to our knowledge.

The current study interrogated the physiological impact of CPAP in healthy young adult rats and investigated the effects of CPAP on CSF flow dynamics and glymphatic-lymphatic transport. Our first step was to create a nasal CPAP device for use in the spontaneously breathing rat and demonstrate that it functioned similarly to clinical devices. After finalizing the chamber design through a series of prototypes, we next performed experiments with the primary goal of pinpointing an optimal positive pressure level that achieved these objectives while avoiding any signs of respiratory distress (tachypnea, irregular breathing patterns). From these experiments, we concluded that slowly increasing the positive pressure to a final value of 3 cmH_2_O yielded the best results as evidenced by expanded upper airway diameter, increased FRC, and improved arterial oxygenation. Therefore, we used a CPAP pressure of 3 cmH_2_O in the DCE-MRI experiments for glymphatic-lymphatic transport assessment.

The DCE-MRI showed that CSF flow speed was significantly increased on the ventral skull base in rats breathing with CPAP compared with controls (Figure 2). Moreover, the velocity flux vectors on the ventral surface were directed toward tissue beds in both groups; however, the vectors were of higher magnitude with CPAP indicating that CSF entered the glymphatic system with inherently higher mean speed. The voxel-wide analysis revealed that CPAP was associated with local increases in glymphatic flux in the dorsal hippocampus, cortex, and olfactory bulb while in the rats breathing via the nose cone, glymphatic transport was increased in the pons and brain stem, suggesting redistribution of glymphatic transport from these areas to the midbrain with CPAP. We also measured the outflow of GadoSpin-P contrast from the brain to the cervical lymph nodes, including the dcLNs. The pattern of lymphatic drainage to the dcLNs, as well as to the accessory and submandibular lymph nodes, was similar between the CPAP and nose cone groups. This suggested that CPAP breathing with a chamber pressure of 3 cmH_2_O did not negatively influence lymphatic drainage.

To identify the mechanisms whereby CPAP enhances CSF flow and glymphatic transport, we specifically targeted known physiological drivers of glymphatic influx, such as vascular pulsatility. It has been known for several decades that vascular pulsatility drives influx of tracers from the subarachnoid CSF compartment into the perivascular space (59, 60). For example, in 1985, Rennels and coworkers showed that transport of a tracer from CSF into the perivascular channels was strikingly diminished by partial ligation of the brachiocephalic arteries (60). The influence of cardiovascular pulsations on perivascular influx was further corroborated with the introduction of the glymphatic system model in 2012, wherein Iliff et al. showed that unilateral ligation of the common carotid artery diminished glymphatic influx ipsilateral to the occlusion (1). Moreover, in another 2-photon microscopy study, arterial pulsatility was measured directly, and systemic administration of the synthetic catecholamine, dobutamine, increased pulsatility as well as glymphatic influx (19). In our study, we did not directly measure cerebral vascular pulsatility but instead tracked the mean ICP and ICP pulse waveform in the CSF compartment via the cisterna magna from rats at baseline and while breathing with CPAP. We first noted that the mean ICP increased approximately 1.7 mmHg from baseline with CPAP. Clinically, CPAP (dependent on its pressure settings) has generally been shown to increase ICP. CPAP is thought to do this through the increase in intrathoracic pressure, which may act as a resistor for venous return from the head. In support of this theory, CPAP has also been shown to increase intraocular pressure in patients with OSA treated with CPAP (61). Further, 15 cmH_2_O CPAP has been associated with a decrease in *spinal* CSF stroke volume and systemic pulse pressure in healthy awake individuals, which is interpreted as a consequence of decreased venous outflow from the calvarium (62).

The ICP recordings in the rats allowed us to analyze the ICP pulse waveform, which in the clinical arena is used to indirectly assess intracranial compliance and can aid in the surgical management of idiopathic normal pressure hydrocephalus (iNPH) (63–65). Interpretation of the mean ICP and ICP waveform is complex as these metrics reflect several physiological variables, such as the arterial pressure, autoregulation, and cerebral venous outflow (64, 65). However, the fundamental component of the ICP pulse pressure waveform has a frequency equal to the HR and as such is a pulsatility metric (65). In our study, analysis of the ICP waveform revealed that the pulse amplitude (but not the respiratory amplitude) was significantly increased in rats breathing with CPAP (Figure 3E). Specifically, the ICP waveform pulse amplitude in the anesthetized rats at baseline was approximately 0.5 mmHg and increased to approximately 0.9 mmHg with 3 cmH_2_O CPAP. To put these numbers into clinical perspective, the rat ICP pulse waveform amplitude (even with CPAP) was several orders of magnitude lower than those reported in humans (63, 66). For example, in a study with intracranial ICP monitoring of patients with suspected iNPH, the *nonelevated* mean ICP pulse pressure waveform amplitude was reported as about 4 mmHg, and the majority of these patients did not undergo shunt surgery, implying that intracranial compliance was normal (63). On the other hand, patients with pulse pressure amplitudes consistently ≥4–6 mmHg during ICP monitoring underwent shunt surgery (63). Although it is difficult to compare mean ICP and pulse pressure waveform amplitude values in small approximately 300 g rats with those observed in humans, we believe that the small-scale ~0.5 mmHg increase in the pulse pressure amplitude with CPAP observed in the rats is unlikely to reflect impaired cerebral compliance and altered cerebral perfusion pressure. Instead, we speculate that the observed increase of the ICP pulse pressure waveform amplitude with CPAP might be beneficial and augment the pulsatile driving force for CSF flow speed and secondarily glymphatic transport. Then again, CPAP might also have acted as a physical stressor and caused release of catecholamines, thereby changing cerebral arterial pulse pressure. Although we did not measure norepinephrine or other endogenous catecholamines, which may have been increased in response to CPAP, the mean arterial blood pressure was unchanged across the 2 groups, suggesting that CPAP did not illicit a stress response. Future clinical and experimental studies should further investigate the potential benefits of CPAP to sustain brain waste clearance in neurodegenerative states, such as aging, which is associated with impaired glymphatic transport (42), sluggish CSF flow, and decreased CSF opening pressure (67).

A number of limitations exist in our study. First, the physiological circumstances of natural sleep were replaced in our study by an anesthetic regimen that resembles sleep physiology in multiple aspects. Thus, dexmedetomidine with supplemental low-dose isoflurane produces electroencephalographic patterns and glymphatic dynamics that resemble those observed with natural non–rapid eye movement sleep (14, 68). Furthermore, although we did not observe overt airway obstruction as seen in clinical OSA, our balanced anesthesia regimen did produce a state wherein the upper airway musculature relaxed, leading to partial collapsibility in the rats breathing via the nose cone. Another limitation of our study, although also addressed to the best of our ability, is our CPAP pressure setting. We attempted to determine an optimal pressure setting based on the most commonly used parameters used in the clinical setting, such as upper airway opening, FRC, and oxygenation, while avoiding respiratory abnormalities. We fine-tuned our setting based on these parameters using separate cohorts; however, we cannot say with certainty that our setting of 3 cmH_2_O is optimal in terms of cerebrospinal and glymphatic dynamics. More studies under different pressure settings will be needed to determine the effects of higher or lower pressures on glymphatic-lymphatic transport function, and most importantly, how the low-pressure CPAP used in the rats might translate to future human studies.

Our study provides insights and an alternative explanation into why CPAP might be associated with beneficial effects on cognitive performance and help slow progression of neurodegenerative diseases in patients with OSA. Although our experiment investigated the effects of CPAP in healthy adult rats that were anesthetized and breathing spontaneously, it serves as an important first step into further research directed at exploring the effects of CPAP on glymphatic-lymphatic fluid dynamics in healthy patients and patients with OSA. As even less invasive techniques of measuring CSF flow dynamics and glymphatic “flow” become available, MRI studies involving natural sleep conducted in humans with and without CPAP may become viable.

## Methods

### Animals and overall study design.

Young adult Sprague-Dawley (SD) female rats (~300 g) purchased from Charles River Laboratories International, Inc. were used for the study. The rats were approximately 8 to 10 weeks old when arriving at Yale University and were allowed at least 1 week to acclimatize prior to experimentation. The rats were housed in an environment with controlled temperature, individually ventilated cages, humidity, and 12-hour light/12-hour cycle from 7 am to 7 pm and were fed standard chow and water ad libitum. All the rats underwent experimentation during their light cycle in a counterbalanced manner across the groups. Our study was divided into different experiments, each designed with unique outcomes objectives outlined in Supplemental Table 2.

### CPAP chamber design.

We designed and tested several CPAP chambers and interface designs (e.g., different configurations and materials for the nasal/snout fitting) on the bench on rats anesthetized with a balanced anesthesia regimen of DEXM-I (13). Both cylindrical and spherical chamber shapes were tested, and in the initial design phase, we permitted both the snout and mouth of the rat to remain inside the chamber, which proved impractical as the seal between the pressurized CPAP device and rat was leaky, and the physical dimensions of the chamber were too big to fit inside the MRI animal holder. As the rat is an obligate nose breather, the nasal CPAP device configuration proved optimal for both positioning and securing a tight seal around the snout. The final CPAP chamber design was compatible with both bench and MRI experiments (Figure 1, A–C, and Supplemental Figure 1). As shown, the CPAP device was designed as spherical chamber with a cylindrical base fitted with a rubber O-ring (inner diameter [I.D.] 13 mm), which facilitated a snug and tight seal around the rat’s snout. The CPAP chamber was 3D printed using acrylonitrile butadiene styrene. The chamber pressure was adjustable by restricting the gas outflow, by means of a plastic valve with barbed fittings (MacMaster-Carr 4757K17), while maintaining the constant gas inflow at 1 L/min. An additional custom 3D printed platform for placing the rat in supine body position in the CPAP or nose cone was designed and fabricated for seamless positioning of the entire ensemble within the homogeneous center of the RF transmit field during MRI experiments (Supplemental Figure 1). The chamber pressure was continuously recorded using an analog nonmagnetic manometer (Dwyer model) connected to a dedicated port in the chamber to ensure that the seal around the rat’s nose was intact while in the MRI scanner. To evaluate the stability of the CPAP chamber pressure, we also performed separate bench experiments with an additional pressure transducer sensor (NXP Semiconductors) to ensure that the chamber pressure was sustained at 3 cmH_2_O for hours (Supplemental Figure 2A). Control rats were breathing via a regular nose cone (Figure 1D) where the gas delivery route is similar to the CPAP chamber, but the outlet did not restrict the outflow and was exposed to atmosphere. The regular nose cone design has been used routinely in previous studies of glymphatic transport (13, 49, 69).

### Measurement of physiological variables in rats with and without CPAP.

All rats were fasted overnight. For anesthesia the rats were induced with 3% isoflurane delivered into an induction chamber in 100% oxygen and then given dexmedetomidine (0.007 mg/kg i.p.) mixed with glycopyrrolate (0.02 mg/kg i.p.). The rats were positioned supine and allowed to breathe spontaneously via the nose cone, and body temperature was controlled at 37°C using a heating pad. Anesthesia was maintained with a subcutaneous infusion of dexmedetomidine (~0.009 mg/kg/h) supplemented with low-dose, approximately 1% isoflurane. However, during surgery, isoflurane was kept at approximately 2% delivered with a FiO_2_ of 30%FiO_2_ of 50%. The fur above the left inguinal area was shaved and cleaned with 70% ethanol solution, a 1 cm incision was made, and the femoral artery was isolated with 4-0 silk sutures. The wound site was infiltrated with 1% lidocaine, and 1 to 2 drops of lidocaine was added to dilate the femoral artery. A PE-50 catheter filled with 0.9% sterile saline solution was inserted into the femoral artery, then secured, and the wound was closed with 4-0 silk. The arterial catheter was flushed with a 10 U/mL heparin/saline solution and hooked up to a pressure transducer (Edwards Baxter IBP disposable pressure transducer), for continuous measurement of blood pressure and pulse pressure during the experiments and for sampling of blood. The surgery for arterial catheterization required approximately 20 to 30 minutes. After placement of the catheter the isoflurane concentration was reduced to 1% and the FiO_2_ to 30%, and the rats were divided into either nose cone or CPAP breathing. For the rats breathing via the nose cone arterial blood gases (ABGs) were taken 3 times at approximately 1 hour, 2 hours, and 3 hours from the time of induction of anesthesia. For the other group, CPAP was gradually implemented by increasing the chamber pressure in small approximately 0.5 cmH_2_O increments every 5 minutes until reaching the final pressure of 3 cmH_2_O and required approximately 30 minutes. In the CPAP group, ABGs were taken at approximately 30 minutes, 90 minutes, and approximately 150 minutes after reaching 3 cmH_2_O CPAP. The total experimental time from the time of induction was approximately 3 hours for both groups, and at the end of the experiments the rats were euthanized as per IACUC-approved protocol guidelines.

### ICP measurements and analysis of ICP waveforms.

For all ICP measurements before and with 3 cmH_2_O CPAP, the rats served as their own controls. Anesthesia with DEXM-I was implemented, and a small CSF catheter was positioned in the cisterna magna with the rat in a stereotaxic frame (9, 13). Subsequently, the rats were transferred to the nasal CPAP device in recumbent position. For all experiments, ICP was measured with a precalibrated pressure sensor probe (Millar’s SPR-1000 Mikro-Tip mouse pressure catheter) inserted into a water-filled 23G stub adapter and sealed by Touhy Borst (70, 71). The ICP probe was zeroed at the level of the head of the rat to determine relative change within each animal when exposed to CPAP. This approach is also used in the clinical setting for ICP monitoring and provides accurate measurements of pressure changes (72). For ICP recording the 23G stub adapter containing the calibrated pressure-sensitive probe was attached to the cisterna magna catheter, and the CPAP chamber was brought to pressure incrementally to the final pressure of 3 cmH_2_O, at which time raw voltage data from the pressure probe were recorded at a frequency of 10 kHz over 5 minutes. Analysis of mean ICP, ICP waveform analysis, and corresponding physiological data were performed in LabChart version 8. Our analysis focused on relative changes from baseline. To best preserve true amplitude, we applied only a low-pass filter at 2 Hz to create the respiratory channel and used cyclic measurements to create maximum and minimum amplitude channels for the respiratory amplitude and created an ICP 2 Hz delta channel. For the pulse waveform, we applied a 45-point Sovitzky-Golay smoothing function to the ICP waveform and then a 5 Hz high-pass filter. We then defined the height (max–min) of this signal as the pulse pressure. For comparisons, we derived the mean of channels at 1-second intervals over 10 respiratory cycles at baseline and at peak CPAP. For each animal, the average ICP respiratory delta and pulse pressure at peak CPAP were divided by their baseline values to derive normalized values. Data from 1 SD rat were used to establish the actual intracranial baseline opening pressure, and the rat did not undergo CPAP.

### Lung volume changes during respiratory cycle measured by MRI.

All rats were fasted overnight, and DEXM-I anesthesia was implemented. The anesthetized rats were transferred to the 9.4 T MRI instrument and placed supine in a custom 3D printed rat body holder as shown in Figure 1C and Supplemental Figure 1. Noninvasive, MRI-compatible monitors (SA Instruments) were used for continuous tracking of RR, HR, and body temperature during the experiment. The overall design for the CPAP experiments conducted in the 9.4 T MRI instrument is illustrated in Supplemental Figure 1A. For CPAP experiments the chamber pressure was continuously recorded using an analog nonmagnetic manometer (Dwyer model) connected to a dedicated port in the chamber to ensure that the seal around the rat’s nose was intact while in the MRI scanner. For temperature control, heated air was delivered into the tube using an MRI-compatible air-heating system (SA Instruments); the body temperature was maintained between 36.5°C and 37.5°C. MRI was performed on a Bruker 9.4T/16 magnet (Bruker BioSpin), operating with Paravision 6.0.1 software, and interfaced with an Avance III-HD console. A commercially available volume transmit-receive RF coil (Bruker BioSpin) with an I.D. of 70 mm was used. Following an anatomical localizer scan ensuring that the whole lung was in the field of view, a respiration-gated 3D FLASH sequence was commenced for lung imaging with the following parameters: repetition time [TR] = 40 ms; echo time [TE] = 3 ms; average = 1; matrix = 128 × 64 × 32; field of view = 100 × 50 × 50 mm (reconstructed at 0.24 × 0.24 × 0.26 mm). Gating pulses were triggered at minimum levels of the respiratory signals for inspiration and expiration as shown in Figure 1H and Supplemental Figure 2, B and C. For these experiments the rats served as their own controls and underwent 2 scans with or without CPAP with the order counterbalanced. At the end of the experiments, the rats were euthanized as per IACUC-approved guidelines.

### DCE-MRI for measurement of glymphatic transport and drainage to the cervical lymph nodes.

All rats were fasted overnight, DEXM-I anesthesia was implemented, and a small catheter made of copper (0.32 mm o.d., Nippon Tockushukan, MFG. CO., LTD) connected to PE-10 tubing was carefully inserted into the cisterna magnum and secured in place using cyanoacrylate glue. The anesthetized rats were transferred to a 9.4 T MRI instrument and placed supine on the custom-built 3D printed animal bed (Figure 1, A–D). During the MRI acquisitions, anesthesia was maintained by 0.8% to 1% isoflurane delivered at an FiO_2_ of 30% along with continuous infusion of dexmedetomidine administered via a subcutaneous route at a rate of 0.009 mg/kg/h. Throughout the experiments, vital signs including pulse oximetry, RR, HR, and body temperature were monitored using noninvasive, MRI-compatible monitors (SA Instruments).

For the DCE-MRI experiments (glymphatic or cervical lymph node drainage), the rats were randomly divided into nose cone and CPAP groups (Figure 1E). For rats breathing with CPAP, the chamber pressure of 3 cmH_2_O was implemented over 30 minutes, after which an anatomical proton density-weighted (PDW) scan and 3 baseline T1-weighted MRI scans were acquired. The same procedure was followed for control rats breathing via the nose cone. For the glymphatic DCE-MRI experiments, 30 μL of 1:10 Gd-DOTA (DOTAREM, Guerbert LLC) diluted in sterile water was infused at a rate of 1.5 μL/min into the CSF after acquisition of baseline data. For the cervical drainage studies, 30 μL of GadoSpin-P was infused into CSF at the rate of 1.5 μL/min using a high-precision pump (LEGATO 130 nanoliter, kd Scientific). For these experiments the lyophilized powder of GadoSpin-P was reconstituted in 850 μL of sterile saline (0.9% NaCl), yielding a 25 mM GadoSpin-P solution, and used within 12 hours of preparation. Imaging was performed on the 9.4 T MRI instrument, and we used a custom-made transmit RF coil (50 mm I.D.) for signal excitation. For the glymphatic experiments, a 3 cm surface RF loop coil (Bruker), positioned under the head of the rat, was used as a receiver, whereas for the cervical lymph node drainage experiments a 2 cm surface RF loop coil (Bruker), positioned over the neck of the rat, was used as a receiver (13) (Figure 4A). Anatomical PDW images at the level of the head were acquired using the 3D FLASH sequence (TR/TE/flip angle [FA]/number of averages = 15 ms/4 ms/7/2, scanning time = 13.5 minutes, resolution = 0.23 × 0.23 × 0.23 mm^3^). Aliasing was prevented by applying a saturation pulse to the extracranial area ventral to the brain. T1-weighted images were acquired using a 3D FLASH sequence with the following parameters: TR/TE/FA/number of averages = 15 ms/4 ms/15°/2, scan time = 5 minutes, resolution = 0.3 × 0.3 × 0.3 mm^3^). For the cervical drainage experiments, T1-weighted images were acquired using a flow compensated 3D FLASH sequence (TR/TE/FA/number of averages = 15 ms/4 ms/15°/1, scanning time = 5.63 minutes, resolution = 0.2 × 0.2 × 0.2 mm^3^). Post-contrast 3D FLASH T1-weighted scans were acquired continuously for 160 minutes and 180 minutes for glymphatic and cervical lymph node drainage experiments, respectively.

### MRI data analysis.

All DCE-MRI data were corrected for head motion and underwent intensity normalization and smoothing with the full-width, half-maximum Gaussian smoothing kernel of 0.1 mm. The smoothened data were used to calculate the voxel-wise percentage signal change from baseline as described previously (14, 49, 73). The percentage signal change from baseline data were used for rOMT analysis (13). Briefly, the mathematical analysis method employed for the DCE-MRI data analysis originates from the theory of OMT, which considers the problem of transporting 1 mass distribution to another with optimality defined via the minimization of a given cost functional (74–76). Here, we used a regularized version of a dynamical OMT model proposed in Benamou and Brenier (76), which we refer to as rOMT. The idea is to add a diffusion term to the original formulation, giving us an advection-diffusion equation, which is more suitable for modeling glymphatic fluid flows (10, 13, 37). More detail on the rOMT algorithm can be found in Supplementary Methods of Chen et al. (13). For the analysis we assume that the percentage signal change from baseline of the DCE-MRI data is proportional to the density function in the rOMT model (37). The 3D images acquired over an approximately 100-minute window were fed into the rOMT model, and the algorithm was run repeatedly between each pair of adjacent images. The returned optimal velocity fields and density functions were subsequently processed with the Lagrangian representation of Glymphatic Dynamics (GLaD) method (37), which traces the trajectories of solutes over time, thereby yielding binary *pathlines*. Important properties such as speed and the norm of the velocity field were derived along the pathlines to form the *speed-(path)lines*. By interpolating the speed-lines into the original voxel resolution, we derived *speed maps*, which can be used to visualize the spatial distribution of solute and fluid transport and speed within the whole brain. From the speed maps, we further derived 2 metrics: (a) the mean solute speed, by taking the average of all positive speed values, and (b) volume transport flux (v-flux), by counting the volume of the pathlines passing through the brain. The mean solute speed measures the overall intensity of the transport, and the v-flux reflects the volume of the pathline network involved. With a given tissue mask compartment (e.g., CSF, brain tissue, the whole brain), we can calculate the 2 metrics for local or global purposes of analysis. To aid the visualization of fluid flow directions, we also derived the velocity flux vectors, which are vectors connecting the starts and endpoints of pathlines. The velocity flux vectors describe how far and in what direction the solute is transported within a certain time window, basically capturing the displacement field. The implementation of the rOMT and the GLaD method was performed with MATLAB_R2022b, and the code is available on GitHub (https://github.com/xinan-nancy-chen/rOMT).

### Normalization of speed maps and voxel-wise statistics.

All the registration and statistical analysis of the processed MRI data were performed in MATLAB-based SPM12 software packages (http://www.fil.ion.ucl.ac.uk/spm). The 3D PDW images were co-registered and resliced to the DCE-MRI data. The co-registered/resliced PDW images were corrected for signal intensity inhomogeneity with the N4 bias field correction algorithm (77). Each bias field–corrected PDW image was then segmented into gray matter, white matter, and CSF tissue probability maps to generate deformation fields using our custom-made tissue probability brain atlas map. The deformation fields were subsequently used to spatially normalize individual pathline speed map data. The isotropic full-width, half-maximum Gaussian smoothing kernel of 0.4 mm was applied to the normalized pathline speed maps. The voxel-wise statistical analysis was performed to compare nose cone versus CPAP smoothed speed maps, using an independent 2-sample *t* test.

### Neck DCE-MRI analysis.

The time series of post-contrast 3D FLASH T1-weighted images from the neck were summed and used as an anatomical template to outline volumes of interest (VOIs) including the CSF compartment and cervical lymph nodes. The CSF VOI was used to normalize the DCE-MRI data for each rat. The TSCs from each of the cervical lymph nodes were extracted from the CSF-normalized DCE-MRI data. The normalized TSC underwent noise cancellation via a 2-time-step moving average analysis using XLSTAT (XLSTAT 2021.1.1, Addinsoft, 2021). The time to peak was extracted from the smoothed normalized TSC of the dcLNs.

### Statistics.

Neither a priori nor a post hoc power analysis was conducted to formally determine or justify sample size due to the unknown effect size of the impact of CPAP on the various outcome metrics when planning the current study. Sample sizes for the DCE-MRI studies were chosen on the basis of similar experiments previously published (10, 13, 38, 49). A linear mixed model with a heterogeneous variance covariance matrix for repeated measures over time was used to analyze the impact of the blood draw time and breathing paradigm on each physiological parameter and within the same rat with fixed effects of time (blood draw), group (nose cone vs. CPAP), and the interaction between time and group. Similarly, a linear mixed models was used with fixed effects of time (respiratory cycle, i.e., end expiration and inspiration), group (no CPAP vs. CPAP), and the interaction between respiratory cycle and group. Group differences were calculated using a post hoc pairwise Fisher’s least significant difference that did not adjust for multiple comparisons. After all the modelings, the least square (marginal) mean difference (and 95% CI) of the outcomes was calculated as the effect size estimate, which would be informative in the design of a future study in which the sample size needs to be directly calculated based on a target statistical power and significance level to detect a prespecified effect size. Comparison of physiological parameters (e.g., HR, RR, pulse pressure), rOMT metrics (e.g., total v-flux, mean CSF speed), or the cervical lymph node studies (e.g., time to peak) across groups was performed using an independent *t* test (2 sided). All the analyses were performed using IBM SPSS Statistics, Version 26. A *P* value of less than 0.05 was chosen to indicate statistical significance, and no adjustment of multiple testing was considered.

### Study approval.

All procedures complied with the Animal Welfare Act, Public Health Service, and the U.S. Department of Agriculture as guided by guided by the U.S. Government Principles for the Utilization and Care of Vertebrate Animals Used in Testing, Research and Training, and they were approved by the IACUC of the Yale Medical School, Yale University.

### Data availability.

See Supporting Data Values.

## Author contributions

BO designed the anesthetic regimen, performed physiological experiments, analyzed and interpreted the data, and helped write the manuscript. SK performed all the brain glymphatic MRI experiments and MRI data analysis. SK and HB designed, performed, and analyzed all MRI experiments of the lymph node drainage. EAB designed the implementation of the CPAP pressure procedure and assisted with the physiological and MRI experiments. XC and AT designed all the computational fluid dynamics algorithms based on rOMT, performed all the rOMT analysis of the data, and wrote the rOMT protocol. ZG designed and performed the ICP measurement experiments. FD assisted with statistical design and data analysis. AL provided intellectual contributions and clinical interpretation of the CPAP data and helped with manuscript writing. PH provided intellectual contributions and performed the ICP waveform analysis. JK provided intellectual contributions and helped interpret the drainage data. HL designed the MRI-compatible CPAP chamber, tested chamber designs and snout/mask interfaces, performed mechanical testing of the CPAP chamber, acquired all the lung data, provided intellectual contributions, and helped write the manuscript. HB conceived the study, designed the experiments, provided intellectual contributions, oversaw data analysis and interpretation, and wrote the manuscript.

## Supplementary Material

Supplemental data

Supporting data values

## Version 1 05/09/2023

In-Press Preview

## Version 2 06/22/2023

Electronic publication
