# Supplementary material for: Continuous positive airway pressure increases CSF flow and glymphatic transport
Source: JCI Insight. 2023 Jun 22;8(12):e170270. doi: 10.1172/jci.insight.170270 (PMC10371231; doi:10.1172/jci.insight.170270)
Supplement: Supplemental data [file jciinsight-8-170270-s050.pdf]

Continuous positive airway pressure (CPAP) increases CSF flow speed and glymphatic transport

Burhan Ozturk, Sunil Koundal, Ehab Al Bizri, Xinan Chen, Zachary Gursky, Feng Dai, Andrew Lim,

Paul Heerdt, Jonathan Kipnis, Allen Tannenbaum, Hedok Lee and Helene Benveniste

Supplemental Figures and Tables

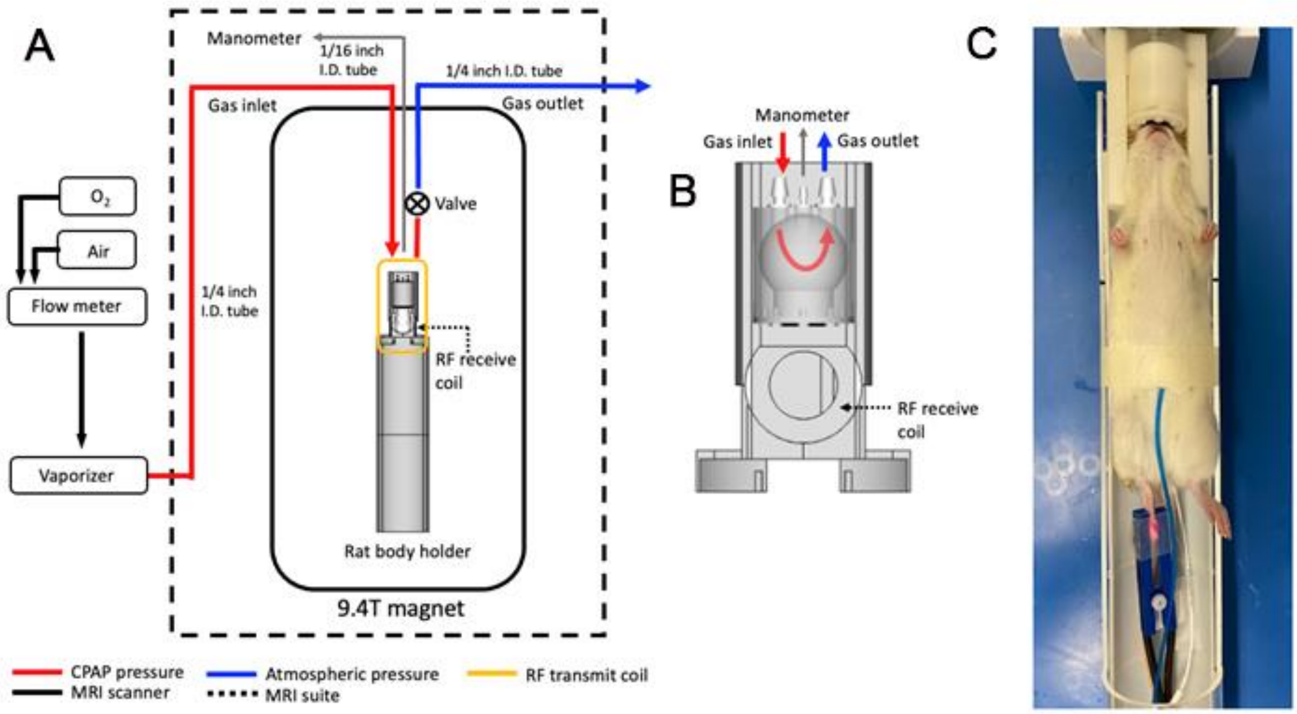

Supplemental Figure 1: Overview of setup for CPAP experiment for MRI or bench experiments.

A Schematic drawing of the experimental setup for CPAP MRI imaging experiments. **B** Drawing of the spherical CPAP chamber with connections to gas in- and outlets in addition to manometer for continuous pressure readings during experiments. **C** Anesthetized rat breathing via the MRI-compatible CPAP chamber. Note that vital sign monitors are in place for continuous measurements of respiratory rate, heart rate, and pulse oximetry.

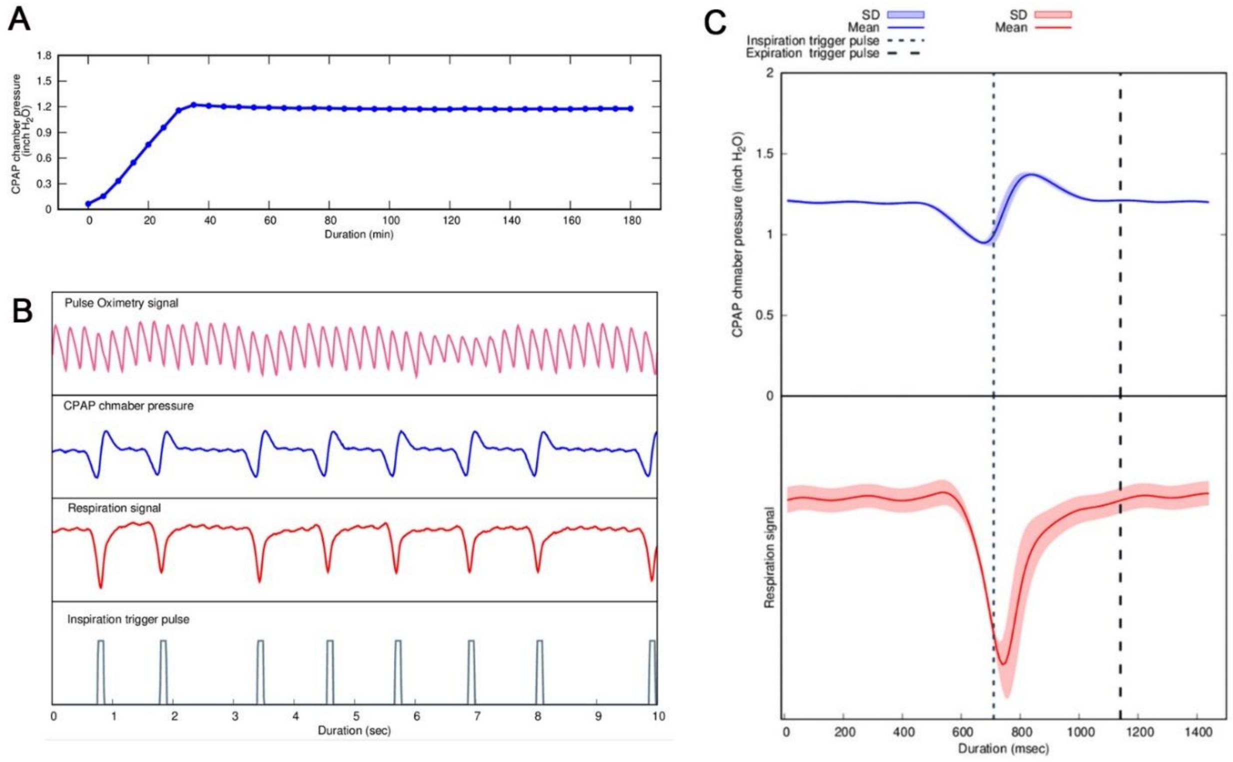

Supplemental Figure 2: Testing of the CPAP chamber pressure and overview of respiratory triggering scheme for MRI lung imaging.

A CPAP chamber pressure as a function of time. Incremental raise in CPAP chamber pressure during the first 30 minutes followed by the steady maintenance of the pressure at 1.2inch H_2O (equal to 3 cmH2O) over 2.5 hours. **B** Representative vital signs (pulse oximetry signal = pink trace, respiratory signal = blue trace) from an anesthetized rat and the corresponding MRI trigger pulse signals (black). The monitored chamber pressure signal (blue trace) was digitized by means of a pressure transducer (NXP Semiconductors). **C** Time averaged (8 minute) CPAP chamber pressure (blue) and respiration signals (red) are shown along with the inspiration and expiration trigger pulse signal shown as dotted lines.

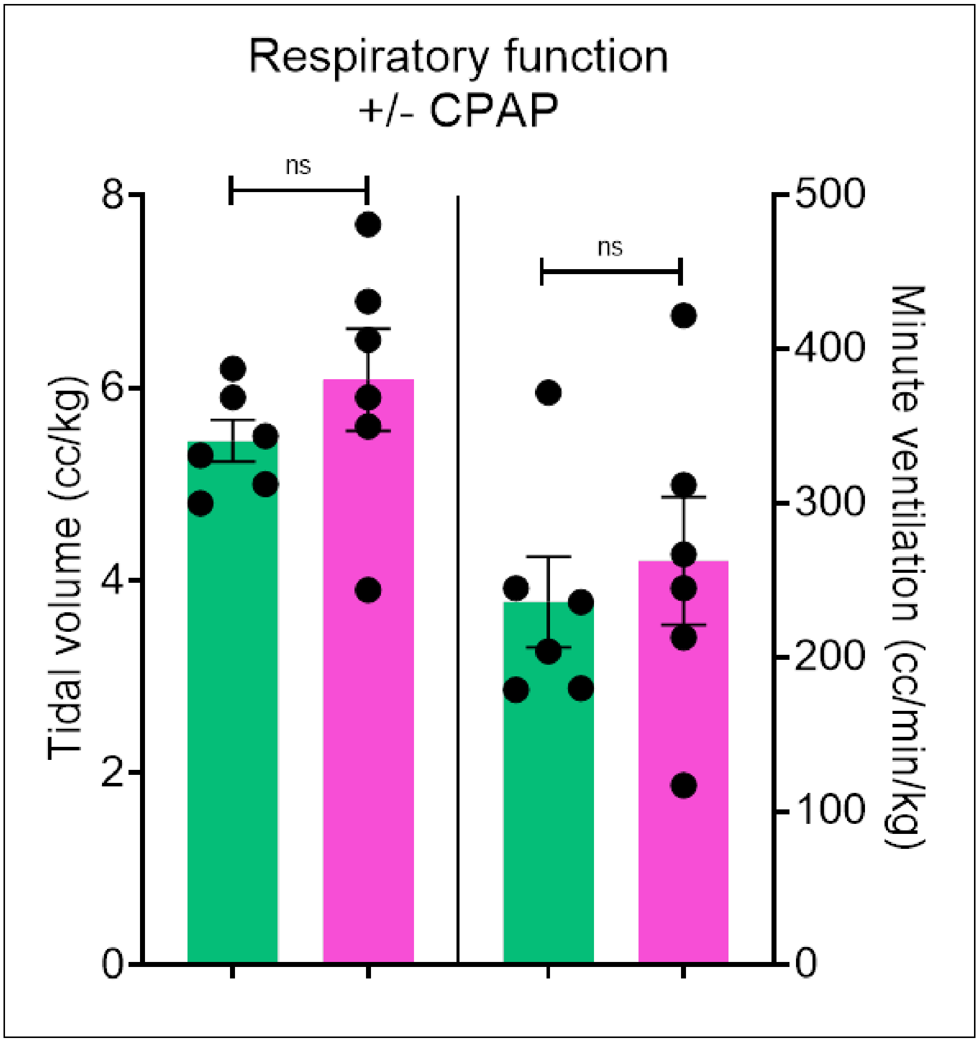

Supplementary Figure 3: Tidal volume and minute ventilation are sustained with CPAP

Graphs with values of tidal volume (defined as the difference between inspiratory volume and functional residual capacity) and the corresponding minute ventilation from rats (n=6) when breathing without CPAP (green bars) or with CPAP (pink bars). Each dot represents values obtained from one rat. Data are mean ± SEM. For these experiments the rats served as their own controls and underwent two different scans with or without CPAP with the order counterbalanced. Statistical analysis for assessing difference between conditions (+/- CPAP) was performed using a paired two-tailed t-test. There were no significant differences in tidal volume (defined as the difference between inspiratory volume and FRC) (p-value = 0.3132) or minute ventilation (p-value = 0.2506) across rats breathing spontaneously with and without CPAP.

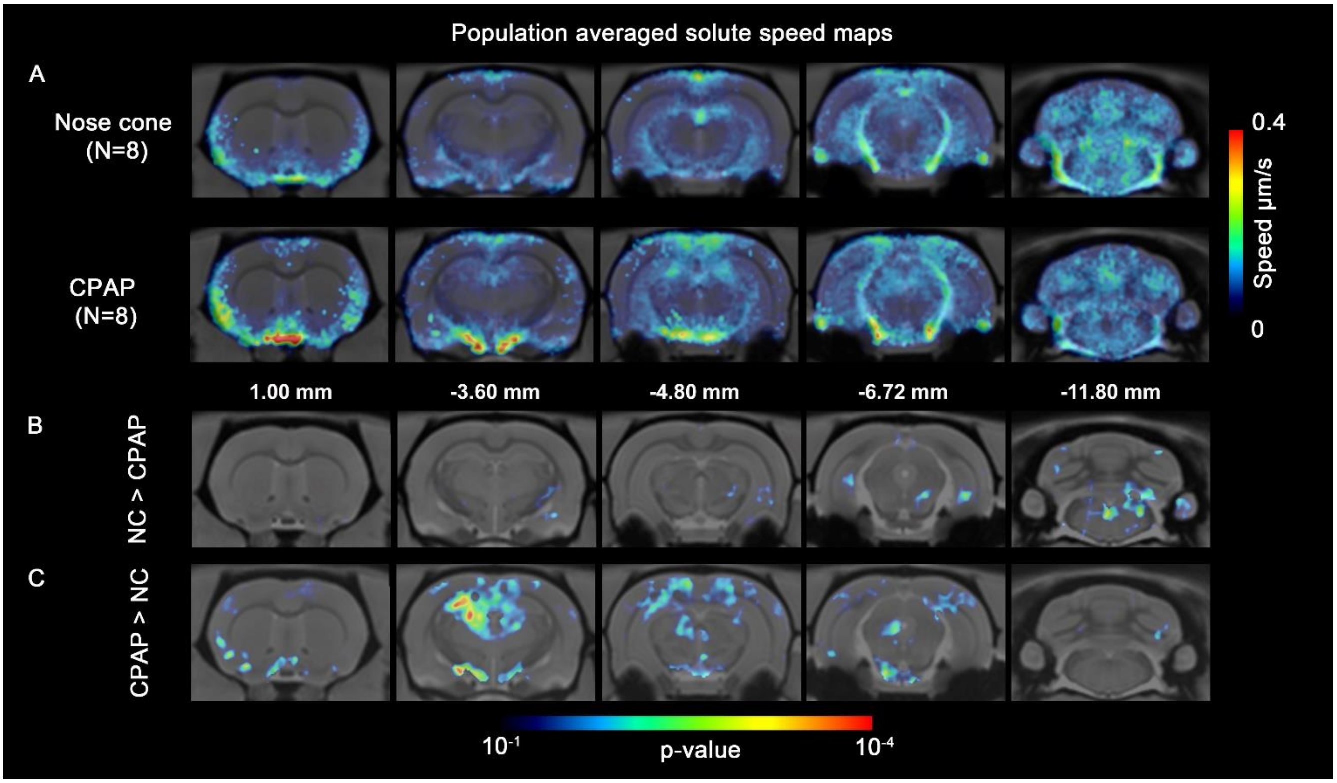

Supplemental Figure 4: Local increase in glymphatic transport observed in rats breathing with CPAP compared to nose cone

A Spatially normalized population-averaged color-coded speed maps of 3-month-old Sprague Dawley (SD) rats breathing via nose cone (N = 8) and age-matched SD rats breathing with CPAP at 3 cmH2O (N=8) are shown overlaid onto population-averaged proton density-weighted anatomical MRI brain templates at different anatomical levels. **B, C** For the control rats breathing via nose cone (N = 8) and CPAP (N = 8) cohorts, statistical parametric maps (color coded for p-values) were calculated at p-value < 0.05 and overlaid onto the MRI brain images to display anatomical areas with significantly higher speed in nose cone (NC) rats in comparison to CPAP rats or the reverse comparison. The p-value map is uncorrected via the false-discovery rate procedure. Anatomical levels of the axially displayed anatomical templates are given by their nearest Bregma distance. Note that increased glymphatic transport in CPAP rats are observed in several areas including the dorsal hippocampus, thalamus, cortical areas.

Supplemental Table 1: Arterial blood gas parameters and arterial blood pressure across groups

		Nose cone (N=6)		CPAP (N=5)		Nose cone vs CPAP				
**Parameter**	Blood draw time (h)	Mean*	SE	Mean	SE	Difference	SE	P-value	L95%	U95%
**PaO_2 (mmHg)**	1	132.5	10.4	154.0	11.4	-21.5	15.5	0.199	-56.6	13.6
	2	112.7	9.3	148.6	10.2	-35.9	13.8	0.028*	-67.1	-4.8
	3	102.0	7.2	170.4	7.9	-68.4	10.7	<0.001**	-92.5	-44.3
**$PaCO_2$ (mmHg)**	1	52.3	2.1	49.0	2.3	3.3	3.1	0.315	-3.8	10.4
	2	51.8	2.9	47.2	3.1	4.6	4.3	0.305	-5.0	14.3
	3	56.5	2.4	53.0	2.6	3.5	3.6	0.350	-4.5	11.5
**pH**	1	7.335	0.019	7.372	0.021	-0.037	0.029	0.232	-0.102	0.028
	2	7.335	0.020	7.334	0.022	0.001	0.030	0.974	-0.067	0.069
	3	7.317	0.024	7.334	0.026	-0.017	0.035	0.634	-0.097	0.062
**Mean arterial Blood Pressure (mmHg)**	1	78.8	3.2	74.0	3.6	4.8	4.8	0.342	-6.1	15.7
	2	73.8	2.4	71.0	2.7	2.8	3.6	0.451	-5.3	11.0
	3	74.2	2.4	70.4	2.6	3.8	3.5	0.315	-4.2	11.8

*Data are presented as least square means and SE's.

**Least square mean differences compare Nose cone vs. CPAP groups at each blood draw. L95%: lower limit of 95% confidence interval (CI), U95%: upper limit of 95% CI.

The mean difference is significant at the 0.05 level.

Supplemental Table 2: Overview of Experimental Groups

Cohorts	Primary purpose	CPAP pressure	Bench experiment	MRI experiment
**SD female rats, Nose cone (N=6) CPAP (N=5)**	Measure arterial blood gases and mean arterial blood pressure	3 cmH_2O	Yes	No
**SD female rats (N=7)**	Measure lung volume +/- CPAP	3 cmH_2O	No	Yes
**SD female rats (N=5)***	Measure intracranial pressure +/- CPAP	3 cmH_2O	Yes	No
**SD female rats Nose cone (N=11)** CPAP (N=11)*****	Measure CSF flow dynamics and glymphatic transport	3 cmH_2O	No	Yes
**SD female rats Nose cone (N=6) CPAP (N=5)**	Measure solute drainage to cervical lymph nodes	3 cmH2O	No	Yes

SD = Sprague Dawley rats, CPAP = Continuous Positive Airway Pressure
*****One rat was excluded from analysis due to drifting baseline
**One rat did not receive CSF contrast (catheter misplacement) and was excluded from the group, 2 rats did not undergo DCE-MRI due to technical issues with the MRI scan.
*******Three rats in the CPAP group were 'failed experiment' either due to dislodgment of the CSF catheter was dislodged and/or technical issues with the set-up.
